# Cutting Edge Therapeutic Insights Derived from Molecular Biology of Pediatric High-Grade Glioma and Diffuse Intrinsic Pontine Glioma (DIPG)

**DOI:** 10.3390/bioengineering5040088

**Published:** 2018-10-18

**Authors:** Cavan P. Bailey, Mary Figueroa, Sana Mohiuddin, Wafik Zaky, Joya Chandra

**Affiliations:** 1 Department of Pediatrics—Research, University of Texas MD Anderson Cancer Center, Houston, TX 77030, USA; cpbailey@mdanderson.org (C.P.B.); mfigueroa1@mdanderson.org (M.F.); 2 The University of Texas MD Anderson Cancer Center, UTHealth Graduate School of Biomedical Sciences, Houston, TX 77030, USA; 3 Center for Cancer Epigenetics, University of Texas MD Anderson Cancer Center, Houston, TX 77030, USA; 4 Department of Pediatrics—Patient Care, University of Texas MD Anderson Cancer Center, Houston, TX 77030, USA; smohiuddin@mdanderson.org (S.M.); wzaky@mdanderson.org (W.Z.)

**Keywords:** pediatric, high-grade glioma, DIPG, therapeutics, epigenetics, clinical trial

## Abstract

Pediatric high-grade glioma (pHGG) and brainstem gliomas are some of the most challenging cancers to treat in children, with no effective therapies and 5-year survival at ~2% for diffuse intrinsic pontine glioma (DIPG) patients. The standard of care for pHGG as a whole remains surgery and radiation combined with chemotherapy, while radiation alone is standard treatment for DIPG. Unfortunately, these therapies lack specificity for malignant glioma cells and have few to no reliable biomarkers of efficacy. Recent discoveries have revealed that epigenetic disruption by highly conserved mutations in DNA-packaging histone proteins in pHGG, especially DIPG, contribute to the aggressive nature of these cancers. In this review we pose unanswered questions and address unexplored mechanisms in pre-clinical models and clinical trial data from pHGG patients. Particular focus will be paid towards therapeutics targeting chromatin modifiers and other epigenetic vulnerabilities that can be exploited for pHGG therapy. Further delineation of rational therapeutic combinations has strong potential to drive development of safe and efficacious treatments for pHGG patients.

## 1. Pathology of Pediatric High-Grade Gliomas and Diffuse Intrinsic Pontine Gliomas (DIPG)

Malignancies of the central nervous system (CNS) have been a constant challenge for clinicians and researchers alike. Due to their complex morphology and relative resistance to therapies, patient mortality is among the highest of all cancers. Pediatric high-grade gliomas (pHGG) constitute about 11% of all pediatric CNS tumors, with an incidence rate of 0.59 per 100,000 [1]. A subset of pHGG are brainstem gliomas, of which the majority are categorized historically as diffuse intrinsic pontine glioma (DIPG) or diffuse midline glioma (DMG), per the new nomenclature. Five-year survival rates for pHGG as a whole are ~28% [1], but are much more dismal for DIPG at ~2% [2]. HGG typically arise from astrocytic origins, including glial, oligodendrocytes, and ependymal cells. These tumors are classified by the World Health Organization (WHO) as either grade III or IV meaning that they are highly malignant tumors with characteristic findings such as hypercellularity, nuclear atypia, and high mitotic activity with or without microvascular proliferation and pseudopalisading necrosis [3]. HGG include a variety of heterogeneous lesions with differing histology, including anaplastic astrocytoma (WHO Grade III), glioblastoma (WHO grade IV) and diffuse midline glioma, H3-K27M mutant (grade IV) as the most common types. Other less common types include anaplastic oligodendroglioma (grade III), anaplastic ganglioglioma (grade III), anaplastic pleomorphic xanthoastrocytoma (grade III), giant cell glioblastoma (grade IV), and gliosarcoma (grade IV) [3,4,5].

## 2. Molecular Alterations of pHGG and DIPG

Efforts to amass tissue specimens from pHGG patients and apply DNA and RNA sequencing technology have reclassified pHGG pathology. Previously, molecular data was not used to classify brain tumors [3], and for pHGG the only parameters known to correlate with patient outcome are extent of surgical resection and histological grade [6,7]. While useful, these measurements cannot inform pre-clinical molecular research or influence targeted therapeutic decisions. The 2016 WHO reclassification of brain tumors [3] incorporates sequencing data for a more complete picture of pHGG pathology. Broadly, pHGG possess mutations in key epigenetic pathways and signaling kinases, and this new understanding of molecular mechanisms has led to clinical trials designed to target these specific vulnerabilities (Table 1).

Early attempts at large-scale profiling of pediatric gliomas utilized comparative genomic hybridization (CGH) techniques that enable detection of deletions and amplifications in a sample with a resolution of approximately 50 kilobases [8]. This study helped confirm that pediatric high-grade gliomas rely less on *EGFR* amplification, which adult grade IV gliomas often possess, and instead rely more on platelet-derived growth factor receptor (*PDGFR*) amplifications. Another new observation was pediatric high-grade gliomas featuring fewer aberrations in the RTK/PI3K/p53/RB “core signaling pathways” seen in adult glioma. A compelling finding was that a distinct phenotypic category (~20% of their cohort) was found to have a “stable genome” with no detectable copy number alterations. This group not only was in stark contrast to the consistently highly-reconfigured genomes of adult gliomas, but featured improved survival over the “amplified” subtypes in the pediatric range [8]. Presence of disease without extensive genomic rearrangement put forth the idea that perhaps very specific and very powerful mutations could potentially be drivers of gliomagenesis and glioma progression. 

Two landmark studies were concurrently published in early 2012 that revealed unique features of pediatric gliomas: they possess mutations in the histone H3 alleles H3.1 (*HIST1H3B*) and H3.3 (*H3F3A*), occurring either at (lysine 27; K to M; H3.1 and H3.3) or near (glycine 34; G to R/V; H3.3 only), a post-translational histone modification point. The K27M variant was found in younger patients (median age, 9 years), midline location including thalamus and brainstem while the G34R/V was more indicative of an adolescent population (median age, 20 years) and hemispheric in location. Due to the co-occurrence of the H3.3 mutation with *TP53* mutations (100% of G34R/V, 82% of K27M) and its conserved nature across patients, this led the authors to believe this is a driver mutation in pHGG [9,10]. These mutations were further found at an even higher prevalence in DIPG, with two independent studies reporting 71% [10] and 78% [11] occurrence of the K27M mutation in a histone H3 variant. Notably, the G34R/V mutation was not found in DIPG, confirming results that it is only seen in supratentorial pHGG. Later findings in 2014 by four separate groups revealed mutations in the activin receptor-like kinase-2 (*ACVR1*) to co-segregate with younger H3.1-K27M, *TP53* wild-type patients whom had slightly improved survival versus other DIPG genotypes [12,13,14,15]. All seven studies mentioned the need for further functional validations of these mutations, and encouraged extensive experimental investigation of their mechanistic impact and role in glioma development. 

Other mutations co-occur with the histone mutations in pHGG, including *ATRX*, *SETD2*, and *TP53* mutations (Table 1). Adult HGG, which do not possess histone mutations, are more likely to have *CDKN2A/B* deletions, *IDH1/2* mutations, *EGFR* amplification and *PTEN* loss. DIPGs follow the pHGG molecular pattern but are further enriched for *TP53* mutation and *PTEN* loss, lack *SETD2* mutations, and possess *PDGFRA* amplification (Table 1) [16,17]. Alterations in cell growth pathways are seen in some pHGGs, including *BRAF* and *FGFR1* mutations and gene fusions among the *TRK* family (*NTRK1/2/3*) (Table 1) [18]; however, *BRAF/FGFR1* perturbations are far more prominent in pediatric low-grade gliomas (pLGGs) [19].

## 3. Histone H3-K27M and G34R/V-Specific Mechanisms in pHGG

A rigorous examination of histone H3 amino acid substitutions using in vitro biochemical assays, HEK-293 cell culture, and murine brain stem glioma models found that the K27M mutation can inactivate the polycomb repressive complex 2 (PRC2) via methionine/isoleucine-specific hydrophobic interactions with the active site of enhancer of zeste homolog 2 (EZH2) [20]. As such, the positive feedback loop regulating PRC2 activity is disrupted and global hypomethylation at H3K27 is observed, along with increased H3K27 acetylation. It was later found that H3.1/3.3-K27M inactivation of PRC2 can also enrich H3K27me3 at specific loci, causing either gene activation (hypo-H3K27me3) or gene suppression (enriched-H3K27me3) in unique pathways [21]. These findings were recapitulated with additional insight into the role of DNA methylation as a consequence of H3.3-K27M, as well as observance of increased H3K27me3 in intergenic regions of the genome, suggesting a possible link with miRNA or lncRNA dysregulation in H3-mutated pHGG [22].

**Figure 1:**
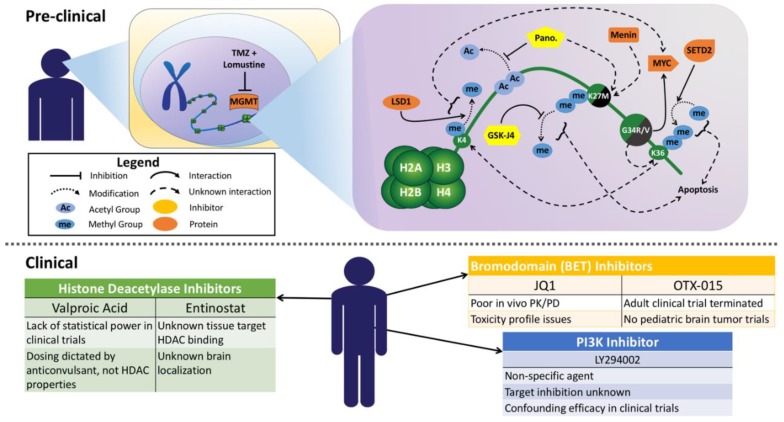
Unexplored pre-clinical molecular interactions and inconclusive clinical data in pediatric high-grade glioma research and trials. Temozolomide (TMZ), O-6-methylguanine-DNA methyltransferase (MGMT), lysine-specific demethylase 1 (LSD1), Pano, MYC, GSK-J4, JQ1, PK/PD, OTX-015, PI3K.

While the K27M mutations possess a potent inhibitory effect on SET-domain containing methyltransferases, the G34R/V mutation did not recapitulate the same effect in vivo at H3K36. However, immunoprecipitated nucleosomes from the same cells showed lowered H3K36me2/3 on the G34R/V-H3.2/3 histone, but not the wild-type H3.2/3 histone in the same oligonucleosome chain [20]. SETD2 is the methyltransferase responsible for generating the H3K36me3 mark, and its interaction with G34R/V mutations has not been extensively studied (Figure 1). The H3.3K36me3 reader protein ZMYND11 cannot bind under G34R/V conditions [23]; however, further investigations of protein complex, reader, and SETD2 binding with G34R/V nucleosomes are needed to provide insight into why the G34R/V mutation does not create a hypomethylated phenotype at the H3K36 residue. 

## 4. Standard of Care Therapies in pHGG including DIPG

The outcome for children with HGG remains dismal despite the use of multi-modal therapy including surgery, radiation therapy (RT) and chemotherapy. In a phase II trial that used temozolomide (TMZ) in combination with lomustine following RT, the authors observed an increase in the overall survival (OS) and event-free survival (EFS) when compared to TMZ + RT alone, but this came with higher toxicities [24]. They hypothesize treatment with multiple alkylating agents can degrade expression of de-alkylating enzymes and increase sensitivity when a second agent is used, but this has not been verified experimentally (Figure 1). Anti-angiogenic therapy with bevacizumab has shown efficacy in recurrent adult glioma [25], but not in newly diagnosed adult glioma patients [26,27] or in combination with lomustine [28]. In pHGG patients, bevacizumab was evaluated in addition to TMZ + RT in patients with newly diagnosed non-brainstem high-grade glioma in a phase II open-label, randomized, multicenter trial (HERBY) [29]. A follow-up molecular analysis of the HERBY trial found addition of bevacizumab to TMZ + RT conferred a survival benefit only in hypermutated and *BRAF*-driven tumors that display an increase in CD8+ T-cell infiltration after therapy [30]. Notably, K27M and G34R/V tumors were “immunologically cold” in their analysis, highlighting how checkpoint blockade and other T-cell-stimulating therapies may not be a viable option for these patients.

DIPG presents a special challenge as their diffuse nature in the brainstem makes surgery not feasible [31,32]. Recent studies have shown MRI-guided stereotactic biopsy to be a safe and reliable method of molecularly characterizing a DIPG patient [33,34], and others were able to detect mutational status using cerebrospinal fluid-sourced tumor DNA [35]. Radiotherapy for DIPG can prolong survival by a few months, but ultimately RT is palliative and patients succumb to progressive disease within a year. Multiple lines of treatment have been tested in clinical trials including chemotherapy and targeted therapy without improving outcome to standard radiation alone [36]. From a clinical therapeutic perspective, it is difficult to discriminate whether the resistance of DIPG to chemotherapy is due to inherent cellular mechanisms or poor delivery of the drug to the tumor site (Table 1). This issue will be addressed later in the review by exploring cutting-edge modalities to improve drug delivery to the brain. 

Studies initially focused on TMZ resistance in pHGG led researchers to the homeobox genes *HOXA9* and *HOXA10*, which were found to be upregulated in pediatric cell lines and correlated to TMZ resistance independent of the demethylating gene; O-6-methylguanine-DNA methyltransferase (*MGMT*) [37]. The pediatric TMZ-resistant cells were also found to be high in progenitor cell markers such as Nestin and CD133, possibly endowing these cells with enhancements in self-renewal and other stem-like properties. Further investigation of the *HOX* signature in adult glioma found that DNA methylation combined with chromosome 7 copy gains endow the adult glioma cells with aberrant *HOX* expression which is normally never seen past the hindbrain [38].

Similar dysregulation of *HOX* expression was also found in adult high-grade astrocytoma and here *HOXA9/10* could be regulated in a PI3K-dependent manner that relied on epigenetic modifications. In their analysis, usage of a PI3K inhibitor decreased *HOX* expression while also increasing trimethylation of lysine 27 on histone H3 (H3K27me3) and decreasing trimethylation of lysine 4 on histone H3 (H3K4me3) [39]. It should be noted the authors used LY294002 to inhibit PI3K, a well-documented non-specific agent [40] that also inhibits CK2, mTOR, and GSK3β [41] along with bromodomains [42] (Figure 1). More recently, targeting of the PI3K pathway in DIPG either with direct chemical agents [43,44] or by modulating the tumor microenvironment [45], has demonstrated efficacy in pre-clinical studies that have not been replicated in a small clinical trial [46]. However, there exists a wide range of drugs designed to target this pathway, and the authors note they did not measure target inhibition in their patient cohort [46]. 

## 5. Histone H3-K27M and G34R/V-Specific Therapies 

With the recent mechanistic insights into how mutant histones poise the epigenome for later tumor development [47], thoughts turned to how to therapeutically target these phenomena and potentially bring new hope to patients with untreatable disease. The well-known oncogene *MYC* was one of the first targets, identified because the G34R/V mutation in H3.3 leads to enrichment of the activating mark H3K36me3 across the genome, with MYC’s genomic locus being the most differentially affected v. H3.3 wild-type [48]. A synthetic lethality screen of 714 kinases in pediatric gliomas identified checkpoint kinase 1 (CHK1) and aurora kinase A (AURKA), both MYC stabilizing proteins, and usage of a small molecule inhibitor of AURKA (VX-689) exhibited potent cell killing in vitro (Table 2). Early neural development genes were also shown to be G34R/V activated, and a later study revealed MYC alone could induce stem-like phenotypes dependent on H3K4 methylation levels regulated by lysine-specific demethylase 1 (LSD1) [49]. Whether the G34R/V mechanism of tumorigenesis is dependent on, concurrent with, or completely independent of the LSD1-MYC interaction remains to be investigated (Figure 1).

Given the role of G34R/V in modulating early neural oncogenesis, it remained to be seen if the K27M mutation would result in a similar fate. Using human embryonic stem cells (hESCs) as a model system, it was observed that transduced H3.3-K27M in combination with p53 silencing and constituently active platelet-derived growth factor receptor A (PDGFRA) conferred neoplastic traits to the cells. A small-molecule screen of >80 epigenetic compounds revealed menin (MEN1) as a potent target specific to K27M-possessing cells [50]. Menin is found in the trithorax histone methyltransferase complex and functions as either a tumor suppressor or an oncogene depending on the cell type. Further study is needed to determine the mechanistic role it plays in H3.3-K27M pediatric glioma as well as a safety/efficacy profile for the MEN1 inhibitor MI-2 [51] (Figure 1, Table 2). Screens on the genetic level using CRISPR-Cas9 have also been used in adult glioma models [61], setting the stage for H3.1/3.3-specific investigation and potential gene therapy applications.

Potential also exists to target epigenetic proteins that participate in the pathways interrupted by H3-K27M-mediated PRC2 inactivation. The hypomethylation signature at H3K27 can potentially be reversed by inhibiting histone H3 demethylases, in particular the ubiquitously transcribed tetratricopeptide repeat, X chromosome (UTX) and Jumonji-domain 3 (JMJD3) demethylases, which are specific to the H3K27 mark. A small-molecule inhibitor of UTX/JMJD3, dubbed GSKJ4, was found to have efficacy in cell lines expressing H3.3-K27M, but displayed no effect in H3.3-G34R/V or wild-type H3.3 controls [52] (Table 2). The exact mechanistic action of the growth inhibition and apoptosis induced by GSKJ4 remains to be explored, as changes in H3K27me2/3 status can broadly affect gene expression (Figure 1). While PRC2 inactivation has been a prime molecular focus of DIPG mutations, the PRC1 complex has recently come to light as a therapeutic target. Single-cell RNA-Seq studies defined the PRC1 subunit BMI-1 to be upregulated in K27M DIPG cancer stem cells with an oligodendrocyte precursor (OPC) phenotype [60]. The authors and others [59] could target BMI-1 with the small molecule PTC-209 and found strong combinatorial effects with *PDGFRA* CRISPR-Cas9 knockouts (Table 2). As such, the more clinically advanced BMI-1 inhibitor PTC-596 is currently in clinical trials for DIPG and pHGG (NCT03605550).

Chromatin-association studies using *Drosophila melanogaster* confirmed the results of Lewis et al. [20], finding H3.3-K27M-containing nucleosomes to be enriched in H3K27 acetylation. A key additional observation was increased presence on these same nucleosomes of bromodomain and extra-terminal motif (BET) proteins BRD1 and BRD4 which function to “read” histone acetylation marks [62]. This suggests use of inhibitors of the BRD family as a therapeutic option in K27M-mutated DIPG, and indeed the pan-BET inhibitor JQ1 has displayed anti-tumor efficacy in pre-clinical models [53,54] (Table 2). Concurrent research examined the use of EZH2 inhibitors, either as a single agent [56] or in combination with BET inhibitors [55]. The mechanism of EZH2 inhibitor tumor suppression was found to be re-expression of p16 and subsequent apoptosis [56], but this could not be recapitulated in a genetically-engineered mouse model of H3.3-K27M DIPG [57] (Table 2). Of note is that the oncogenic driver mutation *BRAF*-V600E, present in a small subset of pHGG, may also benefit from epigenetic therapy. Recent studies have shown *EZH2* to be an oncogene in *BRAF*-driven melanoma [63], and combination therapy of the BRAF inhibitor vemurafinib with JQ1 [64] or DNA methyltransferase inhibitor decitabine [65] displayed efficacy in pre-clinical melanoma models. In clinical studies, a Phase IIa clinical trial of the pan-BET inhibitor OTX-015 in recurrent adult glioblastoma (NCT02296476) was terminated prior to completion, and a pediatric screening trial that includes an EZH2 inhibitor is currently recruiting (NCT03155620). As such, the potential of BET and EZH2 inhibition in pHGG populations remains inconclusive (Figure 1).

High-throughput approaches to find viable “hits” further validated the use of demethylase inhibitors, with GSKJ4 displaying a strong synergistic effect when combined with the pan-histone deacetylase inhibitor (HDACi) panobinostat [66]. As expected, use of these drugs together increased H3 acetylation along with H3K27 methylation. Biochemical analyses of K27M-PRC2 interactions demonstrated that acetylation of H3 at various lysine residues could reverse PRC2 inactivation by over 80-fold [67], but whether this is the main mechanism of panobinostat in K27M models remains to be determined. HDAC inhibitors have previously been shown to be effective against adult glioma [68] and glial stem cells [69], with their efficacy enhanced by inhibiting LSD1. It is currently unknown if dual inhibition of LSD1 (H3K4 demethylation) and UTX/JMJD3 (H3K27 demethylation) can influence HDAC inhibitor-induced cell death, either in adult glioma or H3-mutated pediatric DIPG. 

While Grasso et al. [66] used human xenografts of DIPG, later use of panobinostat in an immune-competent genetically-engineered mouse model of DIPG replicated the in vitro potency of panobinostat but found dose-limiting toxicities when administered in vivo. Panobinostat is well known to cause peripheral thrombocytopenia [70,71], which is not unexpected as it was initially FDA-approved for the blood cancer multiple myeloma. Recent clinical trial data presented at ISPNO 2018 confirms the dose-limiting toxicities of panobinostat when administered peripherally in pediatric patients [72]. Other HDAC inhibitors have been tested in pHGG; however, a retrospective study of valproic acid found no significant enhancement of treatments including radiation and DNA damaging and demethylating agents [73]. Lack of statistical power and dosage of valproic acid as an anticonvulsant, instead of for its HDAC properties, leaves the use of valproic acid in pHGG inconclusive on the clinical end (Figure 1). Given that the only positive in vivo data using HDAC inhibitors in pHGG utilized convection-enhanced delivery directly to the pons [66], alternative delivery strategies of small molecules and biologics to the dense tumor tissue may hold the key to successful pHGG therapy. 

## 6. Therapeutic Delivery

A crucial challenge in glioma drug development is sufficient delivery to the brain, involving passage through the blood-brain barrier (BBB) when a compound is given systemically. Recent work has shown brainstem gliomas have less than one-third the tumor volume and possess lower rates of BBB permeability versus cortical gliomas, independent of H3.3 mutation status [74]. Panobinostat and GSKJ4 were verified in murine brain tissue via paired high-performance liquid chromatography (HPLC)/mass spectrometry (MS) [52,66] but as of yet only panobinostat is in pediatric trials (Table 1). The pharmacokinetics of VX-689, MI-2, OTX-015, EZH2 inhibitors, and PTC-596 in the brain remain unknown, while studies on a multi-kinase drug (BMS-754807) demonstrated efficacy against H3.3-K27M murine cells in vitro but failed to reach IC_50_ levels in tumor tissue when dosed at 50 mg/kg in vivo [58] (Table 2). 

Given these limitations, researchers need to think outside the current small-molecule therapeutic box. One method being explored is focused ultrasound (FUS), which aims to temporarily disrupt the tight junctions in the BBB to allow passage of drugs to the tumor site. It has shown limited efficacy in enhancement of temozolomide treatment [75], and a clinical trial is being pursued using doxorubicin (NCT02343991). This method can also be combined with “microbubbles” to further open the BBB [76], but FUS has not been tested with the epigenetic agents listed above. An even more exciting advancement uses a chain of iron-oxide nanospheres attached to a doxorubicin-loaded liposome. The nanospheres are coated with cyclic arginylglycylaspartic acid (RGD) and targeted to the α_v_β_3_ integrin peptide that is overexpressed in brain tumor vasculature. This flexible “tail” of three iron-oxide spheres increases binding to the endothelial cells, and drug is subsequently released by a localized radio frequency (RF) field that the vibrates the tail and breaks open the liposome [77]. Further studies could adapt this technology to carry other drugs relevant to H3-mutated pediatric tumors, as well as increase drug penetrance to difficult-to-reach areas of the brain such as the pons in DIPG patients.

## 7. Future Directions

While we have explored promising small molecule approaches to treat pHGG, we must consider what role the immuno-oncology revolution will play in pediatric glioma patients going forward. The brain was previously thought to be an immune-privileged site, but this has been proven to not be the case [78]. All cells generate antigens on their cell surface corresponding to internal proteins, and recently researchers were able to isolate K27M-specific T-cells from DIPG patients [79], proving that the immune system can “see” the tumor and generate a reaction specific to its mutational status. Another K27M-driven immunotherapy target in DIPG is the lipid surface marker GD2, for which use of chimeric-antigen receptor T-cells (CAR-T) shows promise in pre-clinical models, but also displays cautionary inflammatory toxicities [80,81]. Furthermore, the potential use of expanded panels of epigenetic agents has been understudied in how it would affect immune function, both systemically and in the tumor microenvironment [82]. There also exists a tight interplay between cellular metabolism and epigenetic functions [83]; there may be other possibilities for modulation of these pathways through nutrient control and metabolic pathway inhibitors. 

A large gap in knowledge of the biology of pHGG is in whether the epigenetic and kinase alterations observed in fully-formed tumors contribute to early oncogenesis of the tumor, or if they are only necessary for mature tumor maintenance. Kinase signaling through FGFR1 [84,85] and PDGFR [86] are crucial to normal CNS development as well as being potentially involved in recovery from radiation-induced necrosis [87], while the role of ACVR1 [88] in CNS function remains more mysterious. For H3.1/3.3 mutations, the highly conserved genotype for age and brain location points to proper H3 modification being involved in gene expression regulation during CNS maturation. It is possible that H3K27 methylation/acetylation is more important infratentorially while H3K36 methylation/acetylation is associated with supratentorial regulation. Complex in vivo models could answer these questions by modifying histones or their associated remodelers over periods of cortical maturation.

It is now understood adult and pediatric gliomas are distinct at the molecular level, a fact that was previously invisible pathologically and can be attributed to the genomics era of low-cost, high-quality sequencing. Rigorous and innovative research has shed new light on how epigenetic and kinase mutations contribute to pHGG molecular signaling. Standard of care currently remains unchanged, but rapidly developing targeted therapies and drug delivery methods are moving into clinical trials and may shift the treatment paradigm of pHGG in the coming years. There are several exciting avenues to pursue in pediatric glioma research, and fresh ideas and new approaches are desperately needed to combat this devastating illness.

## Figures and Tables

**Table 1:** Subtypes of pediatric high-grade gliomas, relevant mutations and features, and pediatric clinical trials designed to target these mutations. Diffuse intrinsic pontine glioma (DIPG), histone H3 K27M variant (H3-K27M), TP53, ACVR1, activin receptor-like kinase-2 (ALK2), ATRX-DAXX, platelet-derived growth factor receptor A (PDGFRA), pan-histone deacetylase (HDAC), FGFR1, MAPK, BRAF, MEK, NTRK, TRK, mTOR, IDH1, MYCN, TERT, SETD2, WEE1, NCT.

Pediatric High-Grade Gliomas: Mutations, Features, and Novel Clinical Therapies				
Anatomical Classification	Defining Mutations	Features	Other Mutations	Targeted Therapies and Pediatric Clinical Trials
Midline location 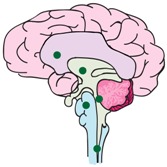	H3-K27M	Includes DIPG Predominantly astrocytic differentiation	TP53 (60%) ACVR1/ALK2 (20–30%) ATRX-DAXX (30%) PDGFRA amplification	H3.3-K27M peptide vaccine ○ NCT02960230 * HDAC inhibitors (Vorinostat, Panobinostat) ○ NCT02420613 ○ NCT01189266 ○ NCT02717455 ○ NCT03566199 PDGFR inhibitors (Crenolanib, Dasatinib) ○ NCT01393912 ○ NCT01644773 ○ NCT00996723
	FGFR1	MAPK activation Usually found in thalamic gliomas		FGFR inhibitor (Erdafitinib) ○ NCT03210714
	BRAF V600E	Rarely co-exists with H3-K27M More common in low grade glioma		BRAF/MEK inhibitors (Dabrafinib, Vemurafanib, Trametinib, Selumetinib, Binimetinib) ○ NCT02684058 ○ NCT01748149 ○ NCT03220035 ○ NCT03363217 ○ NCT01677741 ○ NCT01089101 ○ NCT03213691 ○ NCT02285439
Hemispheric location 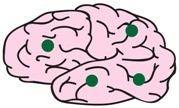	H3-G34R/V	15% Histologically homogenous appearance	ATRX/DAXX (100%) TP53 (90%) NTRK fusion	TRK inhibitors (Larotectinib, Entrectinib) ○ NCT03213704 ○ NCT02637687 ○ NCT03215511 ○ NCT02650401
	IDH1 mutant	Small population Better survival than wild type		mTOR inhibitor (Everolimus) ○ NCT01734512
	H3/IDH wild type		MYCN amplification TP53 PDGFRA TERT	
	SETD2 (15%)	Mutually exclusive with H3-G34R/V		WEE1 inhibitor (Adavosertib)

* All NCT entries indicate trial number listed in clinicaltrials.gov.

**Table 2:** Experimental pre-clinical therapeutics for pediatric high-grade gliomas including compound name, target of therapy, rationale for use in this disease, and references.

Pre-Clinical Therapies for Pediatric High-Grade Gliomas			
Compound(s)	Target	Rationale	Reference
VX-689 (renamed MK-5108)	AURKA (Aurora kinase A)	Destabilizes MYC	[48]
MI-2	MEN1 (Menin)	Blocks menin-MLL-AF9 initiated leukemic oncogenesis; exact role in glioma undefined	[50,51]
GSK-J4	JMJD3 (Jumonji-domain demethylase)	Prevents further demethylation of H3K27 mark in H3-K27M mutated glioma	[52]
OTX-015 JQ1	BRD2/3/4 (Bromodomain-containing proteins)	Interrupts BRD → H3-acetylation binding that is increased by H3.3-K27M	[53,54,55]
GSK126 GSK343 EPZ-6438	EZH2 (Enhancer of zeste homolog 2)	De-represses tumor suppressor p16^INK4a^ and induces apoptosis	[55,56,57]
BMS-754807	Multi-kinase, most potent against IGF-1R (Insulin-like growth factor 1 receptor)	Effective in compound screen; multiple valid targets in DIPG	[58]
PTC-209	BMI-1 (polycomb group RING finger protein 4)	Induces cell cycle arrest and telomerase downregulation, reduces migration, increases sensitivity to radiotherapy	[59,60]
